# Supplementary material for: Curated and harmonised transcriptomics datasets of interstitial lung diseases
Source: Data Brief. 2025 Oct 14;63:112139. doi: 10.1016/j.dib.2025.112139 (PMC12581653; doi:10.1016/j.dib.2025.112139)
Supplement: Supplementary file 1 [file mmc1.zip › Supplementary_material/RNA-seq/fastQC reports/GSE169500/TRIMMED_SRR14057115_fastqc.html]

TRIMMED\_SRR14057115.fastq.gz FastQC Report 

FastQC Report

Sat 3 Dec 2022  
TRIMMED\_SRR14057115.fastq.gz

## Summary

- Basic Statistics
- Per base sequence quality
- Per sequence quality scores
- Per base sequence content
- Per sequence GC content
- Per base N content
- Sequence Length Distribution
- Sequence Duplication Levels
- Overrepresented sequences
- Adapter Content

## Basic Statistics

| Measure | Value |
| --- | --- |
| Filename | TRIMMED\_SRR14057115.fastq.gz |
| File type | Conventional base calls |
| Encoding | Sanger / Illumina 1.9 |
| Total Sequences | 17672974 |
| Sequences flagged as poor quality | 0 |
| Sequence length | 35-369 |
| %GC | 50 |

## Per base sequence quality

## Per sequence quality scores

## Per base sequence content

## Per sequence GC content

## Per base N content

## Sequence Length Distribution

## Sequence Duplication Levels

## Overrepresented sequences

| Sequence | Count | Percentage | Possible Source |
| --- | --- | --- | --- |
| ACACTGAGGACTCTGTTCCTCCCCTTTCCGCCTAGGGGAAAGTCCCCGGA | 302773 | 1.713197790026738 | No Hit |
| GGGAATGTCTACGTGCGTATGCACGTGGCACTCTCTGCCCGAGGTCCGGG | 283605 | 1.604738398868238 | No Hit |
| ACCAATGTCATAGGGTGCAATATCTACAATAGGTAGTCTCACAGCCTTGC | 229246 | 1.297155758843984 | No Hit |
| GGGCCACGAGCTGAGTGCGTCCTGTCACTCCACTCCCATGTCCCTTGGGA | 199840 | 1.1307661064855299 | No Hit |
| AAGCTCAGGGAGAGCCCTGTTAGGGCCGCCTCTGGCCCTAGTCTCAGACC | 183113 | 1.0361187652966615 | No Hit |
| GCTGCTACAGGAGAATAGCAGACAGGTATAGTTAAGAAACAACTTTATTT | 146634 | 0.8297075523338631 | No Hit |
| AGAGCATGACCGATGGATTCCAGTTCGAGTATGGCGGCCAGGGCTCCGAC | 129941 | 0.7352525952904134 | No Hit |
| CAATATCAAGGAAGGGCAGGCGTGATGGCTTATTTGTTTTGTATTCAATG | 129571 | 0.7331590031196786 | No Hit |
| CTAAGTGCTGTGTTGTCGTTCCCCCTGCTTAAAATAAAGTTGTTTCTTAA | 128569 | 0.72748932918704 | No Hit |
| TGGTTGCACGAAACACACTGGGGAATGGAGCAAAACAGTCTTTGAATATC | 124907 | 0.7067684250539835 | No Hit |
| TTCTTGTAGATGGCTGCTCTAAAAAGACAAATGAATGGGGAAAGACAATC | 115645 | 0.6543607204989946 | No Hit |
| ATGTTCTGGGAGGCCTCGGTGGACATCAGGCGCAGGAAGGTCAGCTGGAT | 101484 | 0.5742327239320332 | No Hit |
| TTTACACCTGCTAGGGTGTTCAAAGGTCAGTGCTATAGAAATTCAGTATC | 77882 | 0.44068417686802464 | No Hit |
| AAAGCCAAGAAAACCAACGATGCCAGATACTGAATTTCTATAGCACTGAC | 59539 | 0.33689293041454144 | No Hit |
| ACCCGCAGCTTCTGCTTCTCAGTCAGAAGGTTGTTGTCCTCATCCCTCTC | 47457 | 0.2685286585042223 | No Hit |
| TCTGCAATGCAATCACAATGCCCAAACTAGACCTGCCATTTCTCACACAC | 43045 | 0.24356398645751418 | No Hit |
| TCTGTCTTCTTCAGTTTCGACTTATCGAATTTCTCGATCTCAGCCATATC | 40732 | 0.23047620621181245 | No Hit |
| TTCCCCCTGCGCATGCGGGACTGGCTCAAGAACGTCCTGGTCACCCTGTA | 38992 | 0.22063066465214062 | No Hit |
| TTTTTGTTGCCTGTAAAGTGCTCTGGCATTGCCTGAGGATAGATGAGAAA | 36245 | 0.20508715737373914 | No Hit |
| GGTTATAATCATTGGCAATGTTGTAATCAACAACCCATGTTATTTCTACA | 31901 | 0.18050725361786873 | No Hit |
| TGGTTCCTGGCAAGCCCATGTGTGTTGAGAGCTTCTCAGACTATCCACCT | 31764 | 0.17973205867897502 | No Hit |
| CAGAAGCTCTTTATGTCAAACTTCTCAAGTTGATTGACCTCCAGGTAATA | 31452 | 0.17796665122689592 | No Hit |
| ATTCTTCCTACCCATGAGCATGGAATGTTCTTCCATTTGTTTGTATCCTC | 31332 | 0.17728764836071167 | No Hit |
| TGTTGTGCTAAACGCTGGGAGGGTACAAGTTGGTCATTCCTAAATCTGTG | 30874 | 0.17469612075477506 | No Hit |
| TCACACTTCATGATGGAGTTGAAGGTAGTTTCGTGGATGCCACAGGACTC | 28927 | 0.16367929925093536 | No Hit |
| TCCCTTTCTCCATGCATGAATTTTCAATGCAGACAGCTGTACGATATACG | 28598 | 0.16181769972614682 | No Hit |
| ACACCCACCGCAACTGTCTGTCTCATATCACGAACAGCAAAGCGACCCAA | 28179 | 0.1594468480517201 | No Hit |
| TACTCGTGCGCCTCGCTTCGCTTTTCCTCCGCAACCATGTCTGACAAACC | 27904 | 0.1578907998167145 | No Hit |
| ATTTTCCCAGGTGGGGCCTGCCTGGGGAAAGCTTGTGGCCGGAAGAGAAA | 27690 | 0.15667991137201923 | No Hit |
| AGCGGTTCCGCTGCCCTGAGGCACTCTTCCAGCCTTCCTTCCTGGGCATG | 26792 | 0.15159870659007363 | No Hit |
| CAGTAGCACCAACGTTGATGAGGTCGTGAGTCAGGGGTCCTAGGAAGCTC | 26714 | 0.15115735472705386 | No Hit |
| TGACTGGTGATGCTGATATTGGAGTGCATCTGAGGCTGTTGTAGAAATAA | 25337 | 0.1433657968375894 | No Hit |
| AAAACAGCGTCTTACTGGGGAGGGATATGTGCTTTCTCATCTATCCTCAG | 25164 | 0.1423869010388404 | No Hit |
| ATGTTCTGGGAGCCTCGGTGGACATCAGGCGCAGGAAGGTCAGCTGGATG | 24451 | 0.13835249234226227 | No Hit |
| TGTGCCCTGGAAGCGGAATGGAAACTTGCAGGGCTGTCCTTCAGCGTTGC | 24367 | 0.13787719033593326 | No Hit |
| TCAACGTACTCCATGCCTGGCAGCTTCATCCCGTACTTGTCCACGCACCA | 24181 | 0.13682473589334768 | No Hit |
| TGCGAAAAGTTACTTTCAATGGACTGAACCAGATGATTGTCATAGAACTG | 23887 | 0.1351611788711962 | No Hit |
| ATCGCCTGCGACAAGCCACAGCAGGTGCGCAACATGACGCTGTTCTCCGA | 23581 | 0.13342972156242633 | No Hit |
| TGGCTCTCCCAGAGGCAGAAGGACAATGGCTGTTTCAGGAGCTCTGGGTC | 22729 | 0.12860880121251805 | No Hit |
| AGGATCAGACCATAAAGTGAAGTGAGTCCTTTAAAATCATTTTCTTTGAT | 21914 | 0.12399724007968325 | No Hit |
| AAGAGAAAGCAGTGCAAACCTTCCCGTGGCCGCAAGCGTGGCATCTGCTG | 21899 | 0.12391236472141022 | No Hit |
| CACTTGGCTTCACCCGTGTGACTTTCGTAACGGGGAGAGAGAGAAAAGAT | 21788 | 0.12328428707018978 | No Hit |
| TGAATCTTGAACTGAGTTCCACTTGTAAACTTCTTGTTTCTTGTGGTTCC | 21546 | 0.12191496462338484 | No Hit |
| TGTTCCCTCTCCTCATGAGATTGGTGAAGAAAGTATTTGGCAAAGTTCTT | 21529 | 0.1218187725506754 | No Hit |
| TGTCCTATAACAAGCTTAAAAACATACCAACTGTCAATGAAAACCTTGAA | 21484 | 0.1215641464758563 | No Hit |
| AGAGGCCACAGTAAGGCCCATCCGAGGTCCTGAGGAGATCTTTTCTCTCT | 21234 | 0.12014955717130574 | No Hit |
| ACACTGAGGACTCTGTTCCTCCCCTTTCCGCCTAGGGAAAGTCCCCGGAC | 20910 | 0.11831624943260823 | No Hit |
| ATACTCGAATGCTTGATCTTCAAAACAATAAAATTAAGGAAATCAAAGAA | 20437 | 0.11563984646839859 | No Hit |
| ACACTGAGGACTCTGTTCCTCCCCTTCCGCCTAGGGGAAAGTCCCCGGAC | 19310 | 0.10926287788348468 | No Hit |
| GAGAGGGTCACTTCATCTTCTACTCCTCCCTTTATGGCATTGTTGAGCAG | 19056 | 0.10782565515006133 | No Hit |
| TCTTTCTGGCCTGGAGGCTATCCAGCGTACTCCAAAGATTCAGGTTTACT | 18437 | 0.10432313203199417 | No Hit |
| TCTTCAAGGAGAACTACAAACCACTGCTCAAGGAAATAAAAGAGGATACA | 18079 | 0.10229744014787778 | No Hit |
| TGCATATACTGGTTAGTGAGGTGAGCCTGGCGCTCTTCTTTGCGCTGAGC | 17875 | 0.10114313527536452 | No Hit |

## Adapter Content

Produced by FastQC (version 0.11.7)
